# Benefits of a Mindfulness-Based Intervention upon School Entry: A Pilot Study

**DOI:** 10.3390/ijerph182312630

**Published:** 2021-11-30

**Authors:** Adam Koncz, Ferenc Köteles, Zsolt Demetrovics, Zsofia K. Takacs

**Affiliations:** 1 Doctoral School of Psychology, ELTE Eötvös Loránd University, 1064 Budapest, Hungary; koncz.adam@ppk.elte.hu; 2 Institute of Psychology, ELTE Eötvös Loránd University, 1064 Budapest, Hungary; demetrovics@t-online.hu; 3 MTA-ELTE Lendület Adaptation Research Group, The Hungarian Academy of Sciences, 1064 Budapest, Hungary; 4 Institute of Health Promotion and Sport Sciences, ELTE Eötvös Loránd University, 1117 Budapest, Hungary; koteles.ferenc@ppk.elte.hu; 5 Centre of Excellence in Responsible Gaming, University of Gibraltar, Gibraltar GX11 1AA, Gibraltar; 6 School of Health in Social Science, University of Edinburgh, Edinburgh EH8 9AG, UK

**Keywords:** mindfulness, intervention, school entry, children, stress, cortisol, executive functions

## Abstract

Background: mindfulness meditation is effective at fostering the executive functioning of children, i.e., the skills that play important roles in academic performance and social–emotional wellbeing. One possible mechanism for such an effect might be that meditation practices can decrease stress, especially if someone is at a risk for elevated cortisol levels, for instance, due to a stressful life event, such as starting school. Participants and methods: the present pilot study tested the effects of a six-session mindfulness intervention applied right after school entry compared to a passive control group. In total 61 first graders participated (*M_age_* = 84.95 months, SD = 5.21) in this study from four classes of a primary school in Budapest. Repeated-measures ANOVA were performed to explore the effects on executive functioning skills and cortisol levels. Results: no effect was found on morning salivary cortisol levels, but the working memory capacities of girls significantly improved as a result of the intervention. Conclusions: a relatively short, story-based mindfulness intervention can improve the working memory capacities of first-graders; thus, it could potentially contribute to the academic performance and adaptation of children in schools.

## 1. Introduction

Executive functioning skills are important skills in self-regulation and are necessary for organizing purposeful behaviour [1]. They include three factors: working memory, inhibitory control, and cognitive flexibility [2]. Working memory is used for short-term storage and manipulation of information. In the embedded-process model of Cowan, information, in regard to working memory, originates from the activated subset of long-term storage, which is in the focus of attention; however, the focus of attention is limited [3], which in turn limits working memory. Inhibition is needed to prevent automatic responses that are inappropriate in a situation; finally, shifting skills make us able to flexibly change between rules or look at a problem from different perspectives. Executive functioning skills play an important role in academic performance [4,5]. Actually, the capacity of working memory is found to be the strongest predictor of academic performance in primary education [5]. In addition to school performance, there is a positive association between the developmental stage of executive functions and social-emotional skills [6]. These competencies contribute to the ability of recognizing and managing emotions, to be empathetic and cooperate with others.

Stress experienced in childhood can lead to reduced cognitive performance [7]. It can have a negative impact on the executive functioning of children [8]. There is an inverse relationship between salivary cortisol levels and executive functioning performance. Interestingly, in a meta-analysis that mainly included results from young adults, a positive effect of stress on inhibition—but decreased working memory and cognitive flexibility performance—were found [9]. The authors concluded that this finding supports models proposing that stress drives attention to salient information in order to either avoid or engage with the stressor. School entry is a stressful life event [10]. Starting elementary school requires adjustment to a new environment, novel requirements, and building new social relationships. For example, Groeneveld and colleagues (2013) found higher hair cortisol levels after school entry in a sample of Dutch children [10]. With all of the negative effects of stress in mind, additional efforts to support children in adaptation in this stressful life situation may be beneficial.

One technique to foster children’s executive functioning skills [11] and reduce stress is mindfulness meditation [12,13]. Mindfulness-based interventions (MBI) are becoming very popular for adults and children [14]. The concept of mindfulness is to be in the present moment without judgment [15]. Thus, mindfulness meditation uses different objects to drive the meditator’s attention to the experiences of the present moment, including observation of one’s breathing, bodily sensations, thoughts and emotions, and the environment (e.g., sounds). During mindfulness meditation, meditators are required to monitor their attention and bring it back to the object of the meditation if their minds wander. Mindfulness-based programs are usually very complex, and include a range of different mindfulness practices and often psychoeducational and yoga elements (e.g., mindfulness-based stress reduction, mindfulness-based cognitive therapy). These interventions were found to be beneficial for stress and anxiety in both adults and in youth [16,17,18]. Positive effects were shown on biomarkers of stress levels, such as salivary [12] and blood cortisol levels [19]. These effects are even more pronounced for populations that are at a risk for elevated cortisol levels, for instance, patients with somatic illnesses or people living in stressful life situations [13]. While most of the literature focuses on adult samples, mindfulness-based interventions are more often adapted to (and used with) children [20].

Meta-analytic evidence shows that mindfulness-based interventions are some of the most effective approaches to foster children’s executive functioning skills. Takacs and Kassai (2019) [11] compared the efficacy of the different approaches to enhance children’s executive functioning skills in a series of meta-analyses and found that mindfulness programs represent some of the most promising directions for typically developing children, far exceeding the non-significant effects of physical activity interventions, executive function-specific curricula, or art activities. In fact, benefits of mindfulness-based intervention were comparable to explicit training of executive functioning skills. More specifically, they found significant effects of mindfulness-based interventions on children’s working memory and inhibitory control, but not on cognitive flexibility. It should be noted that these results were based on only six studies assessing the efficacy of mindfulness programs. Dunning and colleagues (2019) [17] also found benefits of mindfulness-based interventions on executive functioning and attention skills of children and adolescents; however, these effects disappeared when compared to active control conditions. Zelazo and Lyons (2012) [21] proposed that mindfulness programs contribute to the development of the executive functioning skills in two ways. First, mindfulness practice improves self-regulation by practicing monitoring and consciously driving one’s attention to the object of the meditation (top-down processing). Secondly, such practice reduces stress, which in turn facilitates cognitive performance (bottom-up processing). The novelty of the present study was to help decide whether a reduction in stress drives the benefits that mindfulness practices have on the executive functioning of children, or if these techniques directly impact executive functions.

While it is a plausible hypothesis that a key mechanism driving the benefits of mindfulness-based interventions on children’s executive functioning skills is stress reduction, the available evidence is equivocal. A mindfulness program called MindfulKids was found effective at preventing stress and behavioural problems in primary school students [22]. Moreover, meta-analyses found that mindfulness-based interventions reduce the symptoms of stress and anxiety in children and adolescents, even compared to active control conditions [17,18]. Concerning results on children’s cortisol levels, Sibinga and colleagues (2013) [23] found that a mindfulness-based stress reduction program protected boys in seventh and eighth grade from low-income families from an increase in cortisol levels. However, another study [24] failed to find such an effect on cortisol levels of fifth-graders.

To test whether the benefits of mindfulness on children’s executive functioning skills are at least partially due to reductions in their stress levels, in a previous experiment, we tested the effects of a short mindfulness-based intervention on executive functioning skills and salivary cortisol when starting school [25]. Contrary to the literature [11,26], we found no effects of mindfulness on children’s executive functioning, which might be due to the fact that the intervention we applied was only five sessions long and perhaps overly intense. All sessions were held within a week. However, we found sex to moderate the effects of the mindfulness program; the intervention prevented a rise in boys’ (but not girls’) cortisol levels after school entry compared to the control group. Based on these findings, we tested a similar, but slightly longer, mindfulness-based program in the present study. We extended the intervention by one session to explain what stress is and why stress management is important to put the intervention in context. Additionally, we applied the intervention right upon school entry for maximal temporal contiguity. To the authors’ knowledge, no studies have tested Zelazo and Lyons’ [21] hypothesis regarding the role of bottom-up processing, in the beneficial effects of mindfulness on children’s self-regulation to date. Additionally, no study has investigated the issue around school entry to date.

In more detail, the following hypotheses were formulated. We assumed that (i) children’s executive functioning skills, especially working memory and inhibitory control skills, could be improved by a mindfulness-based intervention as compared to a passive control group (primary hypothesis). (ii) A mindfulness program after school entry can reduce children’s morning cortisol levels in a stressful life situation, compared to a passive control group. (iii) Based on the model by Zelazo and Lyons [21], a reduction in cortisol levels partially mediates the effects of the mindfulness program, on the executive functioning skills of children.

## 2. Materials & Methods

### 2.1. Design

The study was a randomized pilot study with a “between-subjects” design. First-graders from four classes of a primary school (11, 15, 17, and 18 children) participated. Participants from each class were matched based on sex, age, and pre-test executive function scores, and randomly assigned to either the experimental or passive control group. We chose to randomize participants within the classroom in order to ensure that children were randomly assigned on an individual basis. The first author ensured randomization via a coin flip. Evidently, there was no difference in children’s age (*t*(59) = 0.61, *p* = 0.543) or sex distribution (*X*^2^(1, *N* = 91) = 0.13, *p* = 0.716) between the experimental and the control group (for descriptive statistics, see Appendix A).

### 2.2. Participants

**Figure 1:**
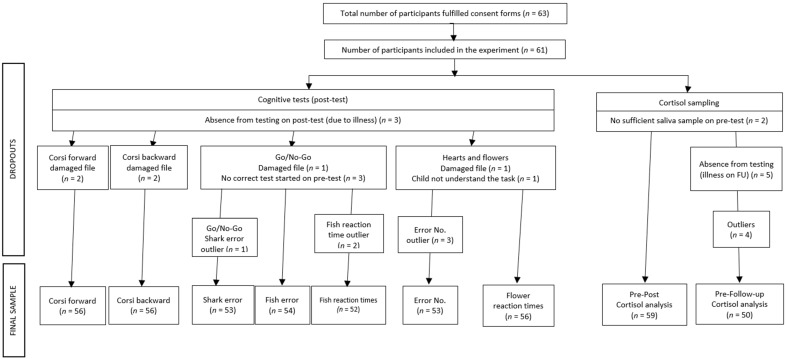
Number of participants whose data could be included in the final analyses.

First-graders from four classes of a state primary school, from an average SES district in Budapest, Hungary, were recruited. A total of 63 parents agreed to participate with their children. Participants with no mental or somatic disorders that could influence children’s cortisol levels were eligible. Two children had to be excluded from the experiment: one because he was a twin sibling of a participant, and another who was missing from school during the pre-tests. Thus, the final sample consisted of 61 children: 31 were assigned to the intervention group (65.6% boys) and 30 to the control group (61.3% boys). Additionally, three participants did not have data on the executive functioning tests because they were missing from school due to illness during the post-test. For a detailed account of the number of participants whose data could be used for the different analyses, see Figure 1. The mean age of the participants was 84.95 months (SD = 5.21), ranging between 73 and 96 months.

### 2.3. Intervention Materials

The mindfulness group attended a six-session-long, story-based mindfulness training over three weeks. A modified and expanded version of a previously used program was applied [25]; (Appendix B). The first session was a modified version of a lesson for children about stress symptoms and opportunities for stress reduction, developed by a Hungarian foundation (Lélekkel az egészségért). The following five sessions were based on a previously used program that was constructed by the authors based on commercially available books for children [25]; (Appendix B). It incorporates mindfulness practices in the storyline that are practiced together with the characters. Another modification for the present study was that we supplemented the program with short questions to the children at the beginning of the sessions, about what they had learned in the previous session, and a discussion at the end of each session for a short summary.

### 2.4. Measurement Instruments

Corsi forward and backward. For measuring children’s short-term and working memory capacity, a computerised version of the Corsi block tapping test was used [27]. During the test, nine squares appeared at different parts of the screen, at a random order. Participants were asked to recall the order, either forward or backward. The test started with two squares flashing as the first level, with one additional square on each level. On each level, there were two items. First, participants were asked to point to the squares that flashed in the same order. This forward version of the test measures short-term memory. After an incorrect answer, the test went back to the previous level and provided two trials on that level. After three errors, the test finished, regardless if it was three consecutive errors or if there were correct trials in between. In the backward version of the test, which measures working memory capacity, the same procedure was followed, except that the child had to point to the squares in reverse order. In both parts of the test, the child received as many points as the number of squares on the highest level they achieved.

Go/No-Go task. A Go/No-Go task was used to measure inhibitory control and sustained attention. In this task, the child had to press a button if a fish appeared on the screen (Go stimulus) but avoid pressing the button if a shark appeared (No-Go stimulus) [28]. Stimuli were presented for 500 ms and the interstimulus intervals were 1200 ms. There were six practice trials after which the children received feedback for their answers. Afterwards, there were 99 trials, two-thirds of which presented the Go stimuli and one-third included the No-Go stimuli. Three scores were computed: the number of errors on the No-Go trials (commission error) measuring inhibitory control, the number of errors on the Go trials (omission error), and the mean reaction time on correct Go trials both measuring sustained attention.

### 2.5. Intervention Materials

The mindfulness group attended a six-session-long, story-based mindfulness training over three weeks. A modified and expanded version of a previously used program was applied [25]; (Appendix B). The first session was a modified version of a lesson for children about stress symptoms and opportunities for stress reduction developed by a Hungarian foundation (Lélekkel az egészségért). The following five sessions were based on a previously used program that was constructed by the authors, based on commercially available books for children [25]; (Appendix B). It incorporated mindfulness practices in the storyline that were practiced together with the characters. Another modification for the present study was that we supplemented the program with short questions to the children at the beginning of the sessions, about what they had learned in the previous session, and a discussion at the end of each session for a short summary.

### 2.6. Measurement Instruments

Corsi forward and backward. For measuring children’s short-term and working memory capacity, a computerised version of the Corsi block tapping test was used [27]. During the test, nine squares appeared at different parts of the screen, at a random order. Participants were asked to recall the order, either forward or backward. The test started with two squares flashing as the first level, with one additional square on each level. On each level, there were two items. First, participants were asked to point to the squares that flashed in the same order. This forward version of the test measures short-term memory. After an incorrect answer, the test went back to the previous level and provided two trials on that level. After three errors, the test finished, regardless if it was three consecutive errors or there were correct trials in between. In the backward version of the test, which measures working memory capacity, the same procedure was followed, except that the child had to point to the squares in reverse order. In both parts of the test, the child received as many points as the number of squares on the highest level they achieved.

Go/No-Go task. A Go/No-Go task was used to measure inhibitory control and sustained attention. In this task, the child had to press a button if a fish appeared on the screen (Go stimulus), but avoid pressing the button if a shark appeared (No-Go stimulus) [28]. Stimuli were presented for 500 ms and the interstimulus intervals were 1200 ms. There were six practice trials, after which, the children received feedback for their answers. Afterwards, there were 99 trials, two-thirds of which presented the Go stimuli and one-third included the No-Go stimuli. Three scores were computed: the number of errors on the No-Go trials (commission error) measuring inhibitory control, the number of errors on the Go trials (omission error), and the mean reaction time on correct Go trials, both measuring sustained attention.

Hearts and Flowers task. The hearts and flowers task [29] was used to assess cognitive flexibility. This test consisted of three blocks. First, hearts appeared on one side of the screen and the participants were asked to press a button on that side (congruent condition). In the second block, flowers appeared on one side of the screen and children were asked to press the button on the opposite side (incongruent condition). Finally, hearts and flowers both appeared in the third block and the task was to follow the previously learnt rules: pressing a button on the same side when hearts were presented and on the opposite side when flowers appeared (mixed condition). This final block measures cognitive flexibility. There were four practice trials at the beginning of the congruent and incongruent blocks and eight practice trials at the beginning of the mixed block, for which the child received feedback. Each block consisted of 40 trials. Stimuli were presented for 1500 ms with a 500 ms long interstimulus interval. Two scores were calculated from the mixed block: the number of errors and the mean reaction times on correct trials.

Baseline cortisol levels. Saliva samples were collected with sponge-ended samplers and stored at −20 °C in Eppendorf tubes until laboratory assessment. Cortisol concentrations (μg/dL) were determined with the method of enzyme-linked immunosorbent assay (ELISA) and the same plate was used to measure all samples of a participant in order to avoid errors caused by inter-assay variation. Two samples were taken at each measurement point (pre-test, post-test, follow-up), and the mean of the two values were used in the statistical analyses.

### 2.7. Procedure

The pre-test was implemented the week before (second week of September) and the post-test—the week after the intervention (third week of October), while a follow-up measurement of salivary cortisol levels was implemented one month after the post-test; that is, during the second week of November. Children were taken from the classroom to an empty room in the school for individual testing sessions, with executive function tests on the pre- and post-test weeks, between 8:00 a.m. and 2:00 p.m. Morning cortisol samples were collected on two consecutive days, between Tuesdays and Thursdays, upon arrival to school from 7:45 a.m. to 8:00 a.m., in all cases/for all measurements (pre-test, post-test and follow-up). Cortisol sampling was timed to mid-week to avoid any differences due to the very first or very last day of the school week.

While participants started the first grade on 2 September, the mindfulness program for the experimental group started during the third week of September. The program consisted of six sessions of 45 min, applied twice a week. Every session was conducted with the experimental group of each class in groups of six to nine children in their own classroom. Overall, there were four mindfulness groups. Trained research assistants who has a minimum of a Psychology BA degree led the sessions under the supervision of a clinical child psychologist. During this time, the control group had free play (i.e., their usual activity) in the schoolyard.

Executive functioning tests were implemented in individual sessions in the school, in designated rooms. All executive functioning tests were performed on a computer and results were recorded by PsychoPy (version 1.85.1) [30]. At the beginning of the testing session, each child received a certificate with his/her name on it, and children earned stickers when they completed a test for the purpose of motivation.

### 2.8. Statistical Analyses

Data screening was carried out before each parametric test. First, outliers (>2 SD) were excluded. Standardized skewness and kurtosis values not exceeding +/−3.29 were considered to reflect normal distribution [31]. In case the assumptions of the planned ANOVA were not met, even after excluding the outliers, as a first step, the square root transformation was performed. If the assumptions were still not met, the non-parametric Mann–Whitney U-test was performed on the untransformed data of the original sample.

To test possible baseline (pre-test) differences on each outcome measure, we planned to run univariate ANOVAs with the pre-test scores on each outcome measure as the dependent variable, condition, and sex as fixed factors. We included the data of those children in these baseline analyses who also had data on the post-test/follow-up and were included in those analyses. Additionally, if a participant was an outlier only on the pre-test results, we excluded him/her only from these baseline analyses.

Hypotheses regarding the effects on executive functioning skills (first hypothesis) and stress levels (second hypothesis) were tested with repeated measures ANOVA for each outcome variable. We used time (pre- to post-test/follow-up) as a “within-subjects” factor, and condition and sex as “between-subject’ factors. Sex was used as a between-subject factor in the analyses because we found children’s sex to moderate the effects of a mindfulness-based program on salivary cortisol levels in a previous study of ours [25].

The third hypothesis about the mediating effect of stress was tested with the SPSS process MACRO [32]. This was tested only in the presence of change in children’s stress levels and cognitive functioning. In this model, the independent variable would be a condition, the mediator the change in cortisol levels, and the dependent variable—the change in cognitive performance.

## 3. Results

### 3.1. Hypothesis I: Improvement of Executive Functions

#### 3.1.1. Corsi Test

##### Short-Term Memory

Regarding the pre-test differences, we used a series of Mann–Whitney U-tests. There was no significant difference between the intervention and control group (*U* = 389.00, *p* = 0.975) or between boys and girls (*U* = 282.00, *p* = 0.162), and no difference between intervention boys and girls (*U* = 66.00, *p* = 0.274) nor control boys and girls (*U* = 72.00, *p* = 0.451). In regard to the effect of the intervention (for descriptive statistics see Table 1), there was no main effects of condition, time, or sex, or any interaction between those on the Corsi forward test results, as shown in Table 2.

##### Working Memory

Two participants’ data had to be excluded on the analyses of the pre-test data because their results were outliers. As shown in Table 3, only the effect of sex was significant on the pre-test scores, showing that girls (*M* = 3.95, SD = 1.18) had higher scores than boys (*M* = 3.03, SD = 1.36) on the pre-test (for further descriptive statistics see Table 1).

Regarding the effects of the intervention, as shown in Table 2, there were no significant main effects of condition or sex on working memory, but there was a significant main effect of time showing that the average score increased from pre-test (*M* = 3.23, SD = 1.48) to post-test (*M* = 3.84, SD = 1.14). No significant condition × sex or time × sex interactions were detected, but there were significant time × condition and time × condition × sex interactions.

To disentangle the time × condition × sex interactions, we ran repeated measures ANOVA, with time as a within-subjects factor and condition as a between-subjects factor, separately for boys and girls. For the boys, there was a significant main effect of time (*F*(1,33) = 9.73, *p* = 0.004, *η*^2^ = 0.228): scores increased from pre-test to post-test regardless of the condition (for descriptive statistics see Table 1. There was no significant main effect of condition (*F*(1,33) = 0.46, *p* = 0.501, *η*^2^ = 0.014) or a time × condition interaction (*F*(1,33) = 0.32, *p* = 0.574, *η*^2^ = 0.010). For girls, there was no significant main effect of time (*F*(1,19) = 2.40, *p* = 0.138, *η*^2^ = 0.112) or condition (*F*(1,19) = 0.65, *p* = 0.429, *η*^2^ = 0.033), but there was a significant time × condition interaction (*F*(1,19) = 9.22, *p* = 0.007, *η*^2^ = 0.327). More specifically, the scores of the girls in the intervention condition increased significantly (*F*(1,9) = 7.86, *p* = 0.021, *η*^2^ = 0.467), while the scores of the girls in the control condition did not change (*F*(1,10) = 1.54, *p* = 0.242, *η*^2^ = 0.134) (for descriptive statistics see Table 1).

#### 3.1.2. Go No/Go Task

Inhibitory control. Regarding the pre-test results, as shown in Table 3, no significant main effect of condition, sex, or interaction between condition × sex were found (for descriptive statistic see Table 1).

When testing the effects of the intervention, there were no main effects of time, condition or sex, and no condition × sex or time × sex or time × condition × sex interactions. However, as shown in Table 2, there was a significant time × condition interactions. Errors in the intervention did not change significantly from pre-test (*M* = 6.41, SD = 4.31) to post-test (*M* = 6.26, SD = 3.88), while the number of errors in the control decreased significantly F(1,24) = 9.05, *p* = 0.006, *η*^2^ = 0.274) from pre-test (*M* = 8.31, SD = 3.81) to post-test (*M* = 6.31, SD = 4.47) (for descriptive statistic see Table 1).

Sustained attention. In regard to the number of omission errors at pre-test, shown in Table 3, there were no significant main effects of time, condition, sex, or condition × sex interactions (for descriptive statistic see Table 1). When testing the effects of the intervention, there were no time, condition, or sex main effects, and no condition × sex, time × condition, time × sex or time × condition × sex interactions (for descriptive statistic see Table 1).

As shown in Table 3 on the pre-test, there were no significant main effects of condition, sex, or condition × sex interactions (for descriptive statistic see Table 1). When testing the effects of interventions (shown in Table 2, there were no significant main effects (time, condition or sex) and no interaction effects (condition × sex, time × condition, time × sex, time × condition × sex) on the reaction time data.

#### 3.1.3. Hearts and Flowers Task

Cognitive flexibility. To analyse pre-test differences, the square root transformation was performed to meet the assumptions of the univariate ANOVA. As shown in Table 3, no significant main effects or interactions were detected.

When testing the effect of intervention, as shown in Table 2, time had a significant main effect: the number of errors decreased from pre-test (*M* = 9.92, SD *=* 7.21) to post-test (*M* = 7.51, SD = 6.94). There were no main effects of condition or sex and no interactions between condition × sex, time × sex, time × condition or time × condition × sex (for descriptive statistic see Table 1).

Sustained attention. Reaction times on correct trials were also analysed. As shown in Table 3, there were no significant effects on the pre-test (for descriptive statistic see Table 1).

When testing the effects of the intervention, as shown in Table 2, there was a marginally significant effect of time: the mean reaction time somewhat decreased from pre-test (*M* = 1.19, SD = 0.36) to post-test (*M* = 1.09, SD = 0.30). No other main effects (condition, sex) or interaction effects (condition × sex, time × condition, time × sex, time × condition × sex) were observed (for descriptive statistic see Table 1).

### 3.2. Hypothesis II: Change in Morning Cortisol Levels

#### 3.2.1. Post-Test Cortisol Levels

Two outliers were excluded from the pre-test results. No significant differences between groups were found (for descriptive statistic see Table 1 and for test statistics see Table 3).

When testing the effects of the intervention on children’s cortisol levels, as shown in Table 4, there were no main effects of condition or sex and no significant condition × sex, time × condition or time × condition × sex interactions. However, a significant effect of time was found. Cortisol levels in participants’ saliva increased from pre-test (*M* = 0.158, SD = 0.115) to post-test (*M* = 0.198, SD = 0.129). There was also a significant time × sex interaction. This interaction was further analysed in repeated measures ANOVA separately for boys and girls. Time was used as a within-subjects factor and condition as a between-subjects factor for both sexes. For boys, there was no significant effect of time (*F*(1,36) = 2.63, *p* = 0.114, *η*^2^ = 0.068) or condition (*F*(1,36) = 0.13, *p* = 0.910, *η*^2^ =< 0.001), and no time × condition interactions were found (*F*(1,36) = 0.000, *p* = 0.992, *η*^2^ =< 0.001) (for descriptive statistic see Table 2). For girls, however, time had a significant effect (*F*(1,20) = 12.98, *p* = 0.002, *η*^2^ = 0.394): cortisol levels raised from pre-test (*M* = 0.145, SD = 0.100) to post-test (*M* = 0.216, SD = 0.122). No effects of condition (*F*(1,20) = 0.16, *p* = 0.900, *η*^2^ = 0.001) or the interaction between time × condition were detected (*F*(1,20) = 0.09, *p* = 0.773, *η*^2^ = 0.004).

#### 3.2.2. Follow-Up Cortisol Levels

Five children were missing from school during follow-up cortisol sampling, and four children had outlying scores on the change from pre-test to follow-up; thus, they were excluded from the following cortisol analyses. Pre-test differences were also tested in this subgroup of participants and, as shown in Table 3, there were no effects of condition or sex and no significant interactions between the (for descriptive statistic see Table 1. When testing the effects of intervention, as shown in Table 4, there were no significant results: no main effect of time, condition or sex, and no interactions between condition × sex, time × condition, time × sex, or time × condition × sex were detected (for descriptive statistic see Table 1).

### 3.3. Hypothesis III: Stress Mediates the Effect on the Executive Functions

We were unable to test whether the reduction in cortisol levels (partially) mediated the effects of the mindfulness program on children’s executive functioning skills because there were no detectable effects of the intervention on cortisol levels.

## 4. Discussion

The present study tested the hypothesis that a reduction in stress levels is one key mechanism driving the benefits of mindfulness-based intervention for children’s executive functioning skills [21]. More specifically, the effects of a six-session long mindfulness-based intervention on first-graders’ executive functioning skills and cortisol levels were investigated on the post-test (one week after finishing the program) and follow-up (one month after the program). There is evidence of mindfulness-based interventions significantly decreasing cortisol levels in adults, but results regarding children are limited and show mixed results [13]. The impact of the program on children’s executive functioning skills, short-term memory, and sustained attention were measured by computerized neurocognitive tasks. This study is the first to test the hypothesis that bottom-up processing, such as reduced stress levels, partially explain the benefits of mindfulness-based interventions on children’s executive functioning skills and contributes to the (very few) studies currently available on the effects of mindfulness on cortisol levels [13] and executive functions [11] in children.

We found no evidence that mindfulness intervention reduced children’s stress levels based on morning cortisol sampling. This finding is in contrast to the results of our previous experiment, showing that a mindfulness program applied before school entry prevented a rise in boys’ stress levels when starting school [25]. Similarly, Sibinga and colleagues (2013) [23] reported a protective effect of a mindfulness-based stress reduction program among boys from low-income families from the seventh and eighth grades against an increase in cortisol levels. Our findings are, however, in line with Schonert-Reichl and colleagues (2015) [24], who did not find any positive effects among fifth-graders. This controversy could be explained by the relatively small effect of the intervention on cortisol level, by the differences between the interventions, or other factors. Further research is needed to shed light on the background of the equivocal findings.

Regarding children’s executive functioning skills, partially in line with previous meta-analytical results of Takacs and Kassai (2019) [11], we found positive effects on working memory capacity for girls, but not for boys. This is in line with the results of Abdi and colleagues (2016) [33], who found a significant effect of an eight-session long mindfulness training on children’s working memory. Additionally, although meta-analytical results showed a significant effect of mindfulness on inhibitory control [11], our results are in line with Abdi and colleagues (2016) [33] and Flook and colleagues (2015) [34], who did not find any positive effects. Interestingly, the opposite was found; the control group showed an improvement in cognitive flexibility. It is important to highlight that interventions included in the meta-analysis were slightly longer (i.e., they included 6–25 sessions). Finally, supporting the meta-analytical results [11], there was no improvement in cognitive flexibility [11]. Moreover, Wimmer and colleagues (2016) found no positive effects in cognitive flexibility in a pilot study [35].

To summarize, while we found some evidence for the efficacy of mindfulness training, results on executive functioning skills depict slightly more nuanced effects. It seems that the mindfulness program made somewhat different gains for the executive functioning skills of boys and girls. It is plausible that this finding is due to girls being more engaged in mindfulness [36]. Additionally, higher school anxiety, neuroticism, and conscientiousness have been found in girls, which may also contribute to better engagement in the intervention and in the tests [37]. The present results are one of the first [25], to our knowledge, to highlight the possible moderating role of individual differences, such as sex in this line of research.

We aimed to measure the effects on cortisol levels in order to test the hypothesis that the positive effects of mindfulness practices on children’s executive functioning skills are, at least partially, due to bottom-up processing, such as reduction in stress, in addition to a top-down effect; that is, mindfulness-based interventions training children’s attention skills [21]. As we found no effects of the intervention on cortisol levels, the present study cannot confirm the assumed role of such bottom-up processing. Instead, it might suggest that top-down processing, such as practicing conscious control over one’s attention during mindfulness practices, plays a more dominant role in the beneficial effects of mindfulness practices in children’s executive functioning. Thus, the overall pattern of our findings does not seem to support the bottom-up component of Zelazo and Lyons’ (2012) [21] model.

In fact, the only finding regarding cortisol levels was that girls experienced an increase from September to October. This result might provide a more fine-tuned account of a previous finding, showing that school entry is a stressful life event [10]. It seems that, from our results, school entry might be especially stressful for girls. It is plausible that girls are under more pressure for good academic achievement and easy social adaptation due to sex stereotyping [38,39,40,41].

Overall, our results might be explained by Eysenck’s Attentional Control Theory of Anxiety [42]. Inhibition, i.e., part of negative attentional control, and cognitive flexibility, i.e., part of positive attentional control, are impaired by anxiety, while there is no association between updating (such as working memory) and the former. This could explain why we did not find any effects on inhibitory control, cognitive flexibility, and stress, but a positive impact on working memory.

Overall, our results suggest that mindfulness does have the potential to help children in the adaptation to school requirements. Although we did not find an effect on children’s stress levels, mindfulness exercises incorporated by teachers around the start of the school year could help to improve children’s executive functioning skills; thus, mindfulness practices can potentially foster later school performance.

## 5. Limitations

This study could be methodologically improved by synchronizing the timing of all cortisol sampling to waking, although these samplings were taken in a narrow time frame: between 7:30 and 8:00 a.m. Further, the sample size was small, especially when testing for possible sex effects and mediation, so non-significant results should be treated with caution. Although randomization was conducted via a coin flip, it was not done by an independent person. Because randomization was done at the individual participant level, members of the control and intervention groups were classmates and might have talked out the sessions. The intervention was somewhat shorter (six sessions) than most commonly used mindfulness programs (about eight sessions). Additionally, this program included not just mindfulness, but psychoeducational content and yoga embedded in a narrative story; thus, it is not clear which component may have had an impact on the participants. It is therefore recommended to repeat the study in the future with a matched active control condition that does not contain mindfulness elements. Moreover, these characteristics of the intervention might make it difficult to compare the results to the findings of previous studies. Finally, a major limitation of the current study was the absence of a manipulation check; that is, we cannot be certain whether we managed to facilitate mindfulness in children. Thus, we cannot exclude the possibility that lack of significant results on cortisol and some measures of executive functioning skills was due to a lack of growth in mindfulness induced during the intervention to start with.

## 6. Conclusions

To summarize, we found no evidence on any effect of mindfulness on children’s cortisol levels, but results partially confirm that mindfulness programs for children are effective at improving working memory. However, this effect was moderated by children’s sex, which should be further investigated in future research. Moreover, the novelty of the present study involved testing the hypothesis that mindfulness practices enhance children’s executive functioning skills by bottom-up mechanisms, such as a reduction in stress [21]. Instead, results point to the importance of top-down processes; that is, practice with monitoring and driving one’s attention consciously.

As far as the practical relevance of the present study—mindfulness-based programs are cheap and no special tools are required to apply them in schools. Thus, they might be a great addition in educational practices; however, further research should focus on how these programs can be carried out by teachers and integrated into “every day” of school.

## Figures and Tables

**Table 1:** Performance on cognitive tests and cortisol levels before and after the intervention (descriptive statistics).

Name of the Outcome	Pre-Test								Post-Test/Follow-Up							
	Intervention				Control				Intervention				Control			
	Boy		Girl		Boy		Girl		Boy		Girl		Boy		Girl	
	Mean (SD)	*n*	Mean (SD)	*n*	Mean (SD)	*n*	Mean (SD)	*n*	Mean (SD)	*n*	Mean (SD)	*n*	Mean (SD)	*n*	Mean (SD)	*n*
**Corsi**																
Forward	3.75 (1.16)	20	4.33 (0.87)	9	3.87 (1.26)	16	4.18 (0.75)	11	3.90 (1.02)	20	4.11 (0.60)	9	4.25 (1.07)	16	4.55 (1.04)	11
Backward	3.20 (1.24)	20	2.90 (1.60)	10	2.80 (1.52)	15	4.18 (1.27)	11	3.80 (1.06)	20	4.30 (0.66)	10	3.67 (1.59)	15	3.73 (0.91)	11
**Go/No-Go**																
Shark (omission) error	6.85 (4.68)	20	5.14 (2.91)	7	7.88 (3.70)	16	9.00 (4.80)	10	6.20 (4.18)	20	6.43 (3.16)	7	6.94 (4.67)	16	5.30 (4.17)	10
Fish (commission) error	44.60 (8.19)	20	45.13 (8.11)	8	45.69 (6.58)	16	42.20 (11.00)	10	42.95 (8.72)	20	39.88 (10.64)	8	41.69 (12.47)	16	43.00 (12.26)	10
Fish reaction time (s)	0.388 (0.024)	18	0.392 (0.040)	8	0.377 (0.036)	16	0.394 (0.024)	10	0.390 (0.027)	18	0.400 (0.036)	8	0.383 (0.035)	16	0.391 (0.017)	10
**Hearts and Flowers**																
Error	11.55 (6.83)	20	10.60 (8.10)	10	9.36 (7.66)	14	6.44 (6.06)	9	8.05 (6.47)	20	7.40 (7.04)	10	7.43 (7.81)	14	6.56 (7.52)	9
Reaction time (s)	1.189 (0.345)	20	1.106 (0.462)	10	1.213 (0.361)	16	1.234 (0.326)	10	1.124 (0.356)	20	1.024 (0.284)	10	1.105 (0.278)	16	1.055 (0.223)	10
**Cortisol**																
Change from pre-test to post-test (μg/dL)	0.173 (0.112)	20	0.151 (0.087)	10	0.160 (0.138)	17	0.139 (0.113)	12	0.190 (0112)	20	0.216 (0.118)	10	0.184 (0.160)	17	0.126 (0.122)	12
Change from pre-test to follow-up (μg/dL)	0.155 (0.094)	18	0.146 (0.096)	8	0.180 (0.144)	14	0.161 (0.112)	10	0.164 (0.083)	18	0.156 (0.096)	8	0.147 (0.132)	14	0.171 (0.131)	10

**Table 2:** Effects of the intervention on short-term memory, working memory and shifting skills.

Measurement	Repeated Measures ANOVA																											
	Time				Condition				Sex				Condition × Sex				Time × Condition				Time × Sex				Time × Condition × Sex			
	*F*	*df*	*p*	*η* ^2^	*F*	*df*	*p*	*η* ^2^	*F*	*df*	*p*	*η* ^2^	*F*	*df*	*p*	*η* ^2^	*F*	*df*	*p*	*η* ^2^	*F*	*df*	*p*	*η* ^2^	*F*	*df*	*p*	*η* ^2^
**Corsi**																												
Forward	0.99	1,52	0.325	0.019	0.64	1,52	0.428	0.012	2.17	1,52	0.147	0.040	0.04	1,52	0.840	0.001	1.05	1,52	0.232	0.027	0.33	1,52	0.570	0.006	0.29	1,52	0.593	0.006
Backward	9.87	1,52	0.003 *	0.160	0.02	1,52	0.886	0.001	1.82	1,52	0.182	0.020	1.04	1,52	0.312	0.020	4.28	1,52	0.044 *	0.076	0.46	0,52	0.500	0.009	7.63	1,52	0.008 *	0.128
**Go/No-Go**																												
Shark (omission) error	2.74	1,49	0.104	0.053	1.08	1,49	0.305	0.021	0.21	1,49	0.648	0.005	0.05	1,49	0.824	0.001	4.75	1,49	0.034 *	0.088	0.12	1,49	0.374	0.002	3.77	1,49	0.058	0.071
Fish (commission) error	3.16	1,50	0.081	0.059	0.000	1,50	0.998	0.001	0.24	1,50	0.629	0.005	0.001	1,50	0.969	0.001	0.42	1,50	0.519	0.008	0.05	1,50	0.834	0.001	2.19	1,50	0.145	0.042
Fish rt	0.77	1,48	0.384	0.016	0.64	1,48	0.429	0.013	1.67	1,48	0.203	0.34	0.14	1,48	0.714	0.003	0.20	1,48	0.661	0.004 *	0.001	1,48	0.970	0.001	0.87	1,48	0.355	0.018
**Hearts and flowers**																												
Errors	9.21	1,49	0.004 *	0.158	1.01	1,49	0.319	0.020	0.48	1,49	0.491	0.010	0.08	1,49	0.780	0.002	3.03	1,49	0.088	0.058	0.70	1,49	0.408	0.014	0.38	1,49	0.538	0.008
Reaction times	3.62	1,52	0.063	0.065	0.22	1,52	0.639	0.004	0.40	1,52	0.529	0.004	0.19	1,52	0.663	0.004	0.52	1,52	0.473	0.010	0.10	1,52	0.755	0.002	0.11	1,52	0.740	0.002

Note. Corsi forward = short-term memory; Corsi backward = working memory; Go/No-Go Shark error = inhibition; go/no-go fish error = sustained attention; Go/No-Go rt. = sustained attention; hearts and flowers errors = cognitive flexibility; hearts and flowers reaction times = sustained attention; *: *p* < 0.05.

**Table 3:** Group (intervention vs. control) and sex differences at pre-test with respect to executive functioning skills and cortisol levels.

Sampling Included on:	Univariate ANOVA											
	Condition				Sex				Condition × Sex			
	*F*	*df*	*p*	*η* ^2^	*F*	*df*	*p*	*η* ^2^	*F*	*df*	*p*	*η* ^2^
**Cortisol**												
Pre-test-Post-test	0.32	1,53	0.574	0.006	0.11	1,53	0.918	<0.001	0.02	1,53	0.884	<0.001
Pre-test-Follow-up	0.35	1,46	0.557	0.008	0.16	1,46	0.687	0.004	0.02	1,46	0.881	<0.001
**Corsi**												
Backward	0.04	1,50	0.836	0.001	5.77	1,50	0.020 *	0.103	1.62	1,50	0.209	0.031
**Go/No-Go**												
Shark (omission) error	3.99	1,49	0.051	0.075	0.06	1,49	0.813	0.001	1.34	1,49	0.252	0.027
Fish (commission) error	0.14	1,50	0.706	0.003	0.37	1,50	0.543	0.007	0.69	1,50	0.411	0.014
Fish reaction time	0.23	1,48	0.637	0.005	1.27	1,48	0.256	0.026	0.58	1,48	0.450	0.012
**Hearts and flowers**												
Mix error	2.40	1,49	0.127	0.047	0.68	1,49	0.412	0.014	0.12	1,49	0.726	0.003
Reaction time	0.54	1,52	0.466	0.010	0.09	1,52	0.762	0.002	0.25	1,52	0.617	0.005

Note. *: *p* < 0.05.

**Table 4:** Effects of the intervention on cortisol levels.

Measurement	Repeated Measures ANOVA																											
	Time				Condition				Sex				Condition × Sex				Time × Condition				Time × Sex				Time × Condition × Sex			
	*F*	*df*	*p*	*η* ^2^	*F*	*df*	*p*	*η* ^2^	*F*	*df*	*p*	*η* ^2^	*F*	*df*	*p*	*η* ^2^	*F*	*df*	*p*	*η* ^2^	*F*	*df*	*p*	*η* ^2^	*F*	*df*	*p*	*η* ^2^
Pre- post	14.06	1,55	<0.001 *	0.204	0.06	1,55	0.811	0.001	0.02	1,55	0.903	<0.001	0.003	1,55	0.954	<0.001	0.13	1,55	0.717	0.002	4.18	1,55	0.046 *	0.071	0.01	1,55	0.915	<0.001
Pre-FU	0.001	1,46	0.971	<0.001	0.10	1,46	0.753	0.002	0.01	1,46	0.931	<0.001	0.04	1,46	0.853	0.001	0.54	1,46	0.466	0.012	0.61	1,46	0.441	0.013	0.57	1,46	0.455	0.012

Note. *: *p* < 0.05.

## Data Availability

The datasets analysed for this study can be found in the Open Science Framework (https://osf.io/ame35/, accessed on 2 July 2021).

## Appendix A

**Table A1:** Descriptive statistics of the control and intervention group who included at least one of the analyses.

	Intervention		Control	
	*n*	Age Months Mean (SD)	*n*	Age Months Mean (SD)
boy	20	85.85 (5.41)	18	82.28 (5.76)
girl	11	84.45 (3.88)	12	83.42 (5.30)
total	31	85.35 (4.90)	30	84.53 (5.56)

## Appendix B

**Table A2:** Schedule of the intervention.

0. Day	1. Day	2. Day	3. Day	4. Day	5. Day
**Discussion about stress, its’ symptoms and stress reduction**	**Breathing meditation** Sea cotter cove **Sensory meditation:** Focusing on sounds **Yoga Postures** **Breathing meditation** Sea cotter cove continued	5. **Muscle relaxation** Angry octopus 6. **Sensory meditation** Touching snail shells 7. **Sensory meditation** Walking meditation	8. **Breathing meditation** Meet again 9. **Sensory meditation** Focusing on sounds 10. **Sitting meditation** Sitting still like a frog	11. **Relaxation story** Bubble riding 12. **Sensory meditation** Mindful eating 13. **Short story** Short summary of the bubble riding story 14. **Sitting meditation** A safe place	15. **Introductory story** 16. **Sitting meditation** The pause button 17. **Short storytelling** 18. **Yoga Postures** 19. **Short storytelling** 20. **Sitting meditation** The conveyor belt of worries 21. **A brief summary of what have been learned during the program**

Sensory and breathing meditation tasks and story elements are based on the Hungarian translations of Lori Lite’s books like Sea Cotter Cove: A Relaxation Story (Lite, 2014) [43], Angry Octopus: An Anger Management Story introducing active progressive muscular relaxation and deep breathing (Lite, 2014) [44], Bubble Riding: A Relaxation Story designed to teach children visualization techniques to increase creativity while lowering stress and anxiety levels (Lite, 2014) [45] and Sensory meditation tasks are inspired by Susan Kaiser Greenland’s Mindful Games: Sharing Mindfulness and Meditation with Children, Teens, and Families (Greenland, 2018) [46]. Sound records of sitting meditations are the modified versions of the Hungarian version of Eline Snel’s books sound records: Sitting Still Like a Frog: Mindfulness Exercises for Kids (and Their Parents) (Snel, 2015) [47]. Some of the yoga postures were based on the Hungarian version of Gilles Diederichs’ book: Playful relaxation −35 relaxing games for children (Diederichs, 2014) [48].
